# Supplementary material for: An enriched network motif family regulates multistep cell fate transitions with restricted reversibility
Source: PLoS Comput Biol. 2019 Mar 7;15(3):e1006855. doi: 10.1371/journal.pcbi.1006855 (PMC6424469; doi:10.1371/journal.pcbi.1006855)
Supplement: S1 Text — (DOCX) [file pcbi.1006855.s001.docx]

**Text S1**

**Enrichment quantification for Type I and Type II motifs in systems without multistep transitions**

We analyzed three published gene regulatory networks (number of nodes 3<n<10, number of edges 8<n<12) that govern dynamical systems with bistable switches, and they are less likely to have multiple intermediate steady states: 1) a network controlling apoptosis [[1](#_ENREF_1),[2](#_ENREF_2)], 2) a network controlling p53 responses [[3](#_ENREF_3)], and 3) a network controlling G1-S transition [[4](#_ENREF_4)]. None of these networks have comparable enrichment of Type I or Type II motifs as in the T cell development network (Table S5).

Although having ‘negative controls’ is important, one has to be cautious about interpreting these observations. Our results imply the positive selection for Type I and Type II motifs because of the importance of multiple attractors in some biological systems, but they do not exclude the possibilities that these motifs were selected for because of some other types of fitness. For example, Ahrends et al. found that the ‘ultra-high feedback systems’ (very similar to our Type I motifs) can produce low cell differentiation rate and zero dedifferentiation rate in the presence noise [[5](#_ENREF_5)]. This performance objective, which depends on bistable switches, is very different from what we found in this paper. Therefore, we do not claim that the enrichment of Type I and Type II is specifically used to achieve the objective of multistep cell fate transitions. Moreover, the three negative control systems are unlikely to produce multiple attractors, and this assumption is based on current knowledge about these systems. It is difficult to conclude that these systems are completely unable or not required to generate the systems containing multiple intermediate states.

**Model equations for early T cell development**

The ODEs for the early T cell development model are listed below.

$$\frac{d\mathrm{TCF}}{dt}=k_{0, T}+K_{T}\frac{{(\mathrm{TCF}/{K_{T,T}})}^{n_{T,T}}}{1+{(\mathrm{TCF}/{K_{T,T}})}^{n_{T,T}}}\cdot\frac{\left( G/{K_{T,G}} \right)^{n_{T,G}}}{1+\left( G/{K_{T,G}} \right)^{n_{T,G}}}\cdot\frac{1}{1+\left( P/{K_{T,P}} \right)^{n_{T,P}}}+k_{T,N}N-r_{d,T}\mathrm{TCF}$$

$$\frac{dP}{dt}=k_{0, P}+K_{P}\frac{{(P/{K_{P,P}})}^{n_{P,P}}}{1+{(P/{K_{P,P}})}^{n_{P,P}}}\cdot\frac{1}{1+\left( G/{K_{P,G}} \right)^{n_{P,G}}}\cdot\frac{1}{1+\left( B/{K_{P, B}} \right)^{n_{P, B}}}\cdot\frac{1}{1+\left( \mathrm{TCF}/{K_{P, T}} \right)^{n_{P, T}}}-r_{d,P}P$$

$$\frac{dG}{dt}=k_{0, G}+K_{G}\frac{{(\mathrm{TCF}/{K_{G,T}})}^{n_{G,T}}}{1+{(\mathrm{TCF}/{K_{G,T}})}^{n_{G,T}}}\cdot\frac{1}{1+\left( P/{K_{G,P}} \right)^{n_{G,P}}}+k_{G,N}N-r_{d,G}G$$

$$\frac{dB}{dt}=k_{0,B}+K_{G}\frac{{(\mathrm{TCF}/{K_{B,T}})}^{n_{B,T}}}{1+{(\mathrm{TCF}/{K_{B,T}})}^{n_{B,T}}}\cdot\frac{{(G/{K_{B,G}})}^{n_{B,G}}}{1+{(G/{K_{B,G}})}^{n_{B,G}}}+k_{G,B}N-r_{d,B}B$$

Here, TCF stands for TCF-1, P stands for PU.1, G stands for GATA3 and B stands for BCL11B. The descriptions and values of the parameters are listed in Table S4.

**Model reduction and performance comparison for the early T cell development**

To reduce the complexity of the system of ODEs and to explore for the 4-attactor systems more efficiently, we took the quasi-steady state assumption for GATA3 and BCL11B. We solved the equations $\dot{G}=0$ and $\dot{B}=0$, and we obtained the steady state levels of GATA3 and BCL11B as the functions $G=S_{G}(TCF, P, \boldsymbol{p})$ and $B=S_{G}(TCF, P, \boldsymbol{p})$. With this approach, the system is reduced two ODEs for TCF-1 and PU.1. Note that this assumption was only used for analyzing steady state behavior, and not for obtaining time course simulation results, stochastic simulations or landscape calculation.

By neglecting the auto-activation of TCF-1 and the activation of TCF-1 by GATA3, the system can be further reduced to one ODE describing dynamics PU.1 using the quasi-steady state assumption. This ODE has the form $\dot{P}=S_{P}\left( P, \boldsymbol{p} \right)-r_{d,P}P$ where$S_{P}$ is a function representing the synthesis rate of PU.1. This reduction was used to construct a continuous objective function for using an optimization procedure to obtain parameter sets that can generate the four-attractor systems. We assumed a hypothetical synthesis rate as a function of PU.1 in the form $S=P-\alpha sin((P+\omega)/\alpha)$. We let $\alpha=6$ and $\omega=0.1$ such that $S$ intersects with the linear degradation function at exactly seven distinct positions for $P\in(0, 3.3)$ (Figure 5B, gray curve). Four of these intersections represent stable steady states (attractors). The objective of finding parameter values that generate four-attractor systems is therefore approximated by a curve-fitting problem in which the function $\sum{{(S}_{P,i}-S_{i})}^{2}$ (sum of squared distance, SSD) is minimized in the domain of $(0, 3.3)$. We used a differential evolution algorithm for the optimization. Optimized parameter sets from two different models were used for comparison of their performance (Figure 5B, purple and red curves), and the SSDs obtained from 500 pairs of optimized parameter sets was used for overall evaluation (Figure 5C). Code for this optimization procedure is available upon request.

**Comparison of the four-stage model with the published bistable model for T cell development**

Manesso et al. has built a model for T cell development with a network of four core transcription factors (TCF-1, PU.1, GATA3 and BCL11B) [[6](#_ENREF_6)]. They fitted their models to a time course of gene expression values, which was inferred from stage-wise expression data obtained from experiments [[7](#_ENREF_7)]. The optimized model suggested an irreversible bistable switch from uncommitted progenitor cells to cells committed to the T cell lineage. Our model used an identical gene regulatory network to study the early T cell development, and we found a large topology (parameter) space that can be responsible for four attractors in the system of development, which is consistent with experimental observations. A key difference between our model and the model by Manesso et al. is that we assumed higher nonlinearity of the regulations. In fact, we found that this assumption is important for the formation of the four-attractor systems, although higher network complexity requires less nonlinearity (Figure S16A). This assumption does not mean that our model requires greater number of binding sites on the promoters of the genes encoding the key TFs, because nonlinearity can arise from multiple mechanisms (for example, Goldbeter-Koshland zero-order ultrasensitivity [[8](#_ENREF_8)], and multi-site phosphorylation [[9](#_ENREF_9),[10](#_ENREF_10)] ), and the Hill exponent is merely a generalized way to describe it [[5](#_ENREF_5)]. Unlike the work by Manesso et al., we did not perform optimization to fit our models to a time course data. Instead, we compared the four attractors obtained from our model to two datasets containing gene expression values of 4 core TFs for the four stages of T cell development, which does not require a conversion from cell states data to time course data. These two datasets differ from each other in terms of absolute values of gene expression, but they both have reasonable agreement with our models after a linear transformation (Figures 9, S7 and S8). The conversion from the stage-wise data to time course data would need a strong assumption of the dynamics of Notch signaling (e.g. whether its strength increases linearly with time). Nonetheless, both models provided very useful insights into the early T cell development and the irreversible nature of the cell fate transitions. More detailed information about the dynamics of the TF concentrations and the biochemical properties of the TFs is necessary to further constrain and improve these models.

**References**

1. Albeck JG, Burke JM, Spencer SL, Lauffenburger DA, Sorger PK (2008) Modeling a snap-action, variable-delay switch controlling extrinsic cell death. PLoS biology 6: e299.

2. Spencer SL, Gaudet S, Albeck JG, Burke JM, Sorger PK (2009) Non-genetic origins of cell-to-cell variability in TRAIL-induced apoptosis. Nature 459: 428.

3. Zhang X-P, Liu F, Cheng Z, Wang W (2009) Cell fate decision mediated by p53 pulses. Proceedings of the National Academy of Sciences 106: 12245-12250.

4. Yao G, Lee TJ, Mori S, Nevins JR, You L (2008) A bistable Rb–E2F switch underlies the restriction point. Nature cell biology 10: 476.

5. Ahrends R, Ota A, Kovary KM, Kudo T, Park BO, et al. (2014) Controlling low rates of cell differentiation through noise and ultrahigh feedback. Science 344: 1384-1389.

6. Manesso E, Kueh HY, Freedman G, Rothenberg EV, Peterson C (2016) Irreversibility of T-cell specification: insights from computational modelling of a minimal network architecture. PloS one 11: e0161260.

7. Mingueneau M, Kreslavsky T, Gray D, Heng T, Cruse R, et al. (2013) The transcriptional landscape of αβ T cell differentiation. Nature immunology 14: 619.

8. Goldbeter A, Koshland DE (1981) An amplified sensitivity arising from covalent modification in biological systems. Proceedings of the National Academy of Sciences 78: 6840.

9. Kapuy O, Barik D, Sananes MRD, Tyson JJ, Novák B (2009) Bistability by multiple phosphorylation of regulatory proteins. Progress in biophysics and molecular biology 100: 47-56.

10. Salazar C, Höfer T (2007) Versatile regulation of multisite protein phosphorylation by the order of phosphate processing and protein–protein interactions. The FEBS journal 274: 1046-1061.
