# Supplementary material for: An enriched network motif family regulates multistep cell fate transitions with restricted reversibility
Source: PLoS Comput Biol. 2019 Mar 7;15(3):e1006855. doi: 10.1371/journal.pcbi.1006855 (PMC6424469; doi:10.1371/journal.pcbi.1006855)
Supplement: S1 Table — (DOCX) [file pcbi.1006855.s002.docx]

**Table S1. Ranges of parameter values for sampling of 3-node networks**

| Parameter | Description | Range/Value |
| --- | --- | --- |
| kA | Maximum regulated production rate of A | 3.3 |
| kA0 | Basal production rate of A | 0.0001 |
| rdA | Effective degradation rate constant of A | 1 |
| nAA | Steepness of the regulation of A by A | [1, 6] |
| KAA | Threshold of the regulation of A by A | [0.05, 4] |
| bAA | Weight of the regulation of A by A | [0, 1] * |
| nAB | Steepness of the regulation of A by B | [1, 6] |
| KAB | Threshold of the regulation of A by B | [0.05, 4] |
| bAB | Weight of the regulation of A by B | [0, 1] * |
| nAC | Steepness of the regulation of A by C | [1, 6] |
| KAC | Threshold of the regulation of A by C | [0.05, 4] |
| bAC | Weight of the regulation of A by C | [0, 1] * |
| kB | Maximum regulated production rate of B | 3.3 |
| kB0 | Basal production rate of B | 0.0001 |
| rdB | Effective degradation rate constant of B | 1 |
| nBA | Steepness of the regulation of B by A | [1, 6] |
| KBA | Threshold of the regulation of B by A | [0.05, 4] |
| bBA | Weight of the regulation of B by A | [0, 1] * |
| nBB | Steepness of the regulation of B by B | [1, 6] |
| KBB | Threshold of the regulation of B by B | [0.05, 4] |
| bBB | Weight of the regulation of B by B | [0, 1] * |
| nBC | Steepness of the regulation of B by C | [1, 6] |
| KBC | Threshold of the regulation of B by C | [0.05, 4] |
| bBC | Weight of the regulation of B by C | [0, 1] * |
| kC | Maximum regulated production rate of C | 3.3 |
| kC0 | Basal production rate of C | 0.0001 |
| rdC | Effective degradation rate constant of C | 1 |
| nCA | Steepness of the regulation of C by A | [1, 6] |
| KCA | Threshold of the regulation of C by A | [0.05, 4] |
| bCA | Weight of the regulation of C by A | [0, 1] * |
| nCB | Steepness of the regulation of C by B | [1, 6] |
| KCB | Threshold of the regulation of C by B | [0.05, 4] |
| bCB | Weight of the regulation of C by B | [0, 1] * |

* The sum of the weights for each target TF is 1.
