# Supplementary material for: An enriched network motif family regulates multistep cell fate transitions with restricted reversibility
Source: PLoS Comput Biol. 2019 Mar 7;15(3):e1006855. doi: 10.1371/journal.pcbi.1006855 (PMC6424469; doi:10.1371/journal.pcbi.1006855)
Supplement: S3 Table — (DOCX) [file pcbi.1006855.s004.docx]

**Table S3. Experimental evidence supporting the regulations in the early T cell development model**

| Regulation | References |
| --- | --- |
| TCF1 auto-activation | [[1](#_ENREF_1)] |
| TCF1 inhibits PU.1 | [[2-4](#_ENREF_2)] |
| PU.1 inhibits TCF1 | [[2-4](#_ENREF_2)] |
| TCF1 activates BCL11B | [[1](#_ENREF_1)] |
| TCF1 activates GATA3 | [[1](#_ENREF_1),[5](#_ENREF_5)] |
| GATA3 activates TCF1 | [[1](#_ENREF_1),[5](#_ENREF_5)] |
| GATA3 activates BCL11B | [[5](#_ENREF_5)] |
| GATA3 inhibits PU.1 | [[3](#_ENREF_3),[6](#_ENREF_6),[7](#_ENREF_7)] |
| PU.1 inhibits GATA3 | [[3](#_ENREF_3),[6](#_ENREF_6),[7](#_ENREF_7)] |
| PU.1 auto-activation | [[8-10](#_ENREF_8)] |
| BCL11B inhibits PU.1 | [[1](#_ENREF_1),[5](#_ENREF_5)] |
| Notch activates TCF1 | [[1](#_ENREF_1),[11](#_ENREF_11)] |
| Notch activates GATA3 | [[3](#_ENREF_3),[12-14](#_ENREF_12)] |
| Notch activates BCL11B | [[2](#_ENREF_2),[15](#_ENREF_15)] |
| Notch influences TCF1, GATA3 and BCL11B | [[1](#_ENREF_1),[4](#_ENREF_4),[11](#_ENREF_11),[12](#_ENREF_12),[16](#_ENREF_16)] |

**References**

1. Weber BN, Chi AW, Chavez A, Yashiro-Ohtani Y, Yang Q, et al. (2011) A critical role for TCF-1 in T-lineage specification and differentiation. Nature 476: 63-68.

2. Franco CB, Scripture-Adams DD, Proekt I, Taghon T, Weiss AH, et al. (2006) Notch/Delta signaling constrains reengineering of pro-T cells by PU.1. Proc Natl Acad Sci U S A 103: 11993-11998.

3. Del Real MM, Rothenberg EV (2013) Architecture of a lymphomyeloid developmental switch controlled by PU.1, Notch and Gata3. Development 140: 1207-1219.

4. Schmitt TM, Zuniga-Pflucker JC (2002) Induction of T cell development from hematopoietic progenitor cells by delta-like-1 in vitro. Immunity 17: 749-756.

5. Garcia-Ojeda ME, Klein Wolterink RG, Lemaitre F, Richard-Le Goff O, Hasan M, et al. (2013) GATA-3 promotes T-cell specification by repressing B-cell potential in pro-T cells in mice. Blood 121: 1749-1759.

6. Yui MA, Feng N, Rothenberg EV (2010) Fine-scale staging of T cell lineage commitment in adult mouse thymus. J Immunol 185: 284-293.

7. Taghon T, Yui MA, Rothenberg EV (2007) Mast cell lineage diversion of T lineage precursors by the essential T cell transcription factor GATA-3. Nat Immunol 8: 845-855.

8. Okuno Y, Huang G, Rosenbauer F, Evans EK, Radomska HS, et al. (2005) Potential autoregulation of transcription factor PU.1 by an upstream regulatory element. Mol Cell Biol 25: 2832-2845.

9. Leddin M, Perrod C, Hoogenkamp M, Ghani S, Assi S, et al. (2011) Two distinct auto-regulatory loops operate at the PU.1 locus in B cells and myeloid cells. Blood 117: 2827-2838.

10. Zarnegar MA, Rothenberg EV (2012) Ikaros represses and activates PU.1 cell-type-specifically through the multifunctional Sfpi1 URE and a myeloid specific enhancer. Oncogene 31: 4647-4654.

11. Tydell CC, David-Fung ES, Moore JE, Rowen L, Taghon T, et al. (2007) Molecular dissection of prethymic progenitor entry into the T lymphocyte developmental pathway. J Immunol 179: 421-438.

12. Taghon TN, David ES, Zuniga-Pflucker JC, Rothenberg EV (2005) Delayed, asynchronous, and reversible T-lineage specification induced by Notch/Delta signaling. Genes Dev 19: 965-978.

13. Weerkamp F, Luis TC, Naber BA, Koster EE, Jeannotte L, et al. (2006) Identification of Notch target genes in uncommitted T-cell progenitors: No direct induction of a T-cell specific gene program. Leukemia 20: 1967-1977.

14. Van de Walle I, De Smet G, De Smedt M, Vandekerckhove B, Leclercq G, et al. (2009) An early decrease in Notch activation is required for human TCR-alphabeta lineage differentiation at the expense of TCR-gammadelta T cells. Blood 113: 2988-2998.

15. Li P, Burke S, Wang J, Chen X, Ortiz M, et al. (2010) Reprogramming of T cells to natural killer-like cells upon Bcl11b deletion. Science 329: 85-89.

16. Germar K, Dose M, Konstantinou T, Zhang J, Wang H, et al. (2011) T-cell factor 1 is a gatekeeper for T-cell specification in response to Notch signaling. Proc Natl Acad Sci U S A 108: 20060-20065.
