# Supplementary material for: An enriched network motif family regulates multistep cell fate transitions with restricted reversibility
Source: PLoS Comput Biol. 2019 Mar 7;15(3):e1006855. doi: 10.1371/journal.pcbi.1006855 (PMC6424469; doi:10.1371/journal.pcbi.1006855)
Supplement: S4 Table — (DOCX) [file pcbi.1006855.s005.docx]

**Table S4. Parameter values for early T cell development model**

| Parameter | Description | Value | Unit |
| --- | --- | --- | --- |
| kT0 | Basal production rate of TCF-1 | 0.48 | A.U./6 hours |
| kT | Regulated production rate of TCF-1 | 1.12 | A.U./6 hours |
| rdT | Effective degradation rate constant of TCF-1 | 1 | /6 hours |
| nTT | Steepness of the activation of TCF-1 by TCF-1 | 4 | Unitless |
| nTP | Steepness of the inhibition of TCF-1 by PU.1 | 6 | Unitless |
| nTG | Steepness of the activation of TCF-1 by GATA3 | 6 | Unitless |
| kTN | Strength of the inhibition of TCF-1 by Notch | 1 | A.U./6 hours |
| KTT | Threshold of the activation of TCF-1 by TCF-1 | 0.5 | A.U. |
| KTP | Threshold of the inhibition of TCF-1 by PU.1 | 3.5 | A.U. |
| KTG | Threshold of the activation of TCF-1 by GATA3 | 0.4 | A.U. |
| kP0 | Basal production rate of PU.1 | 0.32 | A.U./6 hours |
| kP | Regulated production rate of PU.1 | 2.88 | A.U./6 hours |
| rdP | Effective degradation rate constant of PU.1 | 1 | /6 hours |
| nPT | Steepness of the inhibition of PU.1 by TCF-1 | 2 | Unitless |
| nPP | Steepness of the activation of PU.1 by PU.1 | 6 | Unitless |
| nPG | Steepness of the inhibition of PU.1 by GATA3 | 4 | Unitless |
| nPB | Steepness of the inhibition of PU.1 by BCL11B | 3 | Unitless |
| KPT | Threshold of the inhibition of PU.1 by TCF-1 | 2.5 | A.U. |
| KPP | Threshold of the activation of PU.1 by PU.1 | 0.62 | A.U. |
| KPG | Threshold of the inhibition of PU.1 by GATA3 | 2.5 | A.U. |
| KPB | Threshold of the inhibition of PU.1 by BCL11B | 2.6 | A.U. |
| kG0 | Basal production rate of GATA3 | 0 | A.U./6 hours |
| kG | Regulated production rate of GATA3 | 2.2 | A.U./6 hours |
| rdG | Effective degradation rate constant of GATA3 | 1 | /6 hours |
| nGT | Steepness of the activation of GATA3 by TCF-1 | 2 | Unitless |
| nGP | Steepness of the inhibition of GATA3 by PU.1 | 6 | Unitless |
| kGN | Strength of the inhibition of GATA3 by Notch | 0.02 | A.U./6 hours |
| KGT | Threshold of the activation of GATA3 by TCF-1 | 0.2 | A.U. |
| KGP | Threshold of the inhibition of GATA3 by PU.1 | 2.2 | A.U. |
| kB0 | Basal production rate of BCL11B | 0 | A.U./6 hours |
| kB | Regulated production rate of BCL11B | 5.5 | A.U./6 hours |
| rdB | Effective degradation rate constant of BCL11B | 1 | /6 hours |
| nBT | Steepness of the activation of BCL11B by TCF-1 | 2 | Unitless |
| nBG | Steepness of the activation of BCL11B by GATA3 | 6 | Unitless |
| kBN | Strength of the inhibition of BCL11B by Notch | 0.06 | A.U./6 hours |
| KBT | Threshold of the activation of BCL11B by TCF-1 | 1.9 | A.U. |
| KBG | Threshold of the activation of BCL11B by GATA3 | 1.5 | A.U. |

* A.U. stands for arbitrary unit for concentrations.
