# Supplementary figures and images for: An enriched network motif family regulates multistep cell fate transitions with restricted reversibility
Source: PLoS Comput Biol. 2019 Mar 7;15(3):e1006855. doi: 10.1371/journal.pcbi.1006855 (PMC6424469; doi:10.1371/journal.pcbi.1006855)

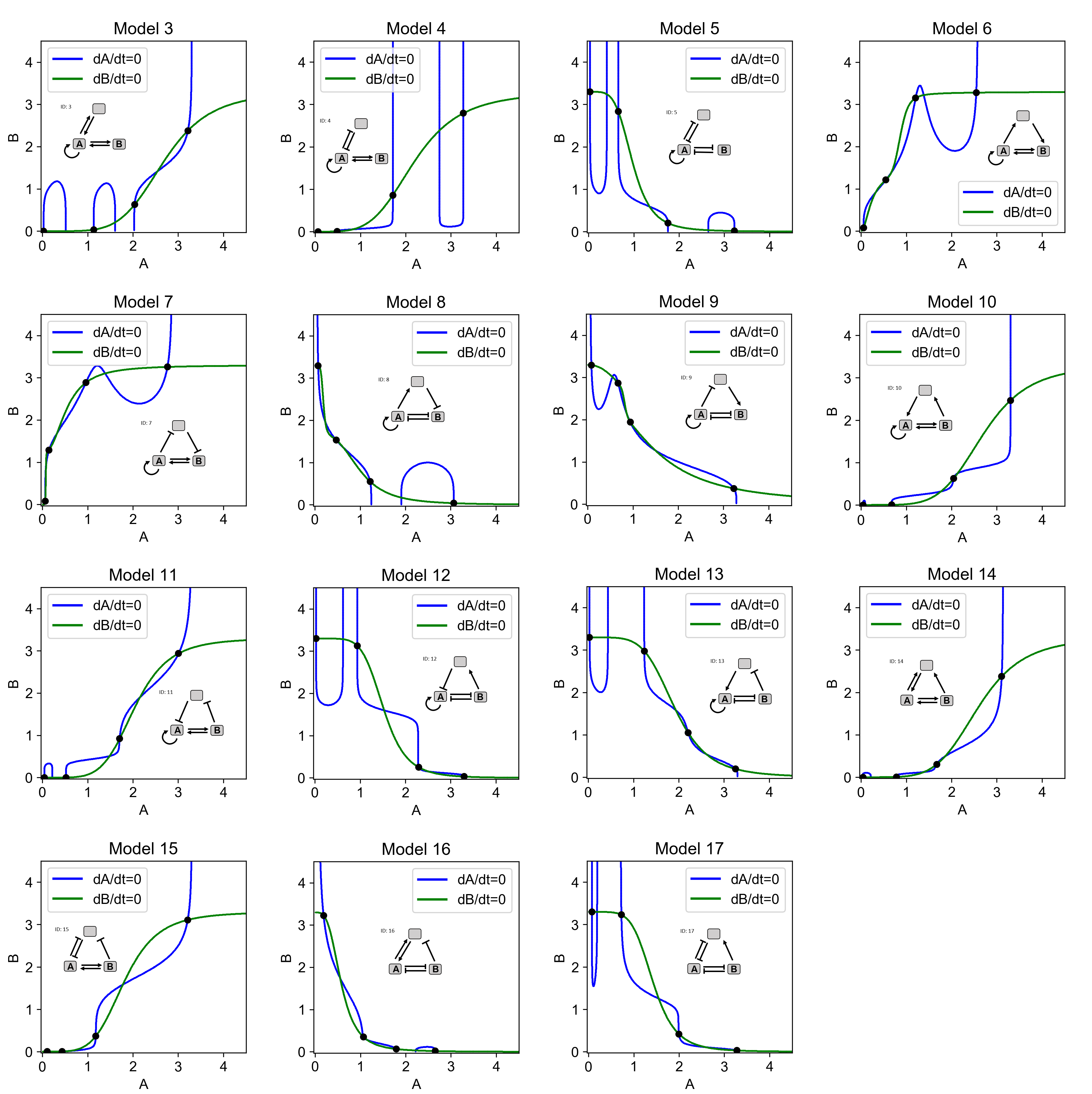

Supplement: S1 Fig — Nullclines for TF A (the node on the left of the network diagram) and TF B (the node on the right of the network diagram) are shown. Stable steady states are shown as black dots. The inset network diagram shows the corresponding network. Random parameter sampling was used to obtain the parameter sets that allows the 4-attractor systems. (TIF) [file pcbi.1006855.s007.tif]

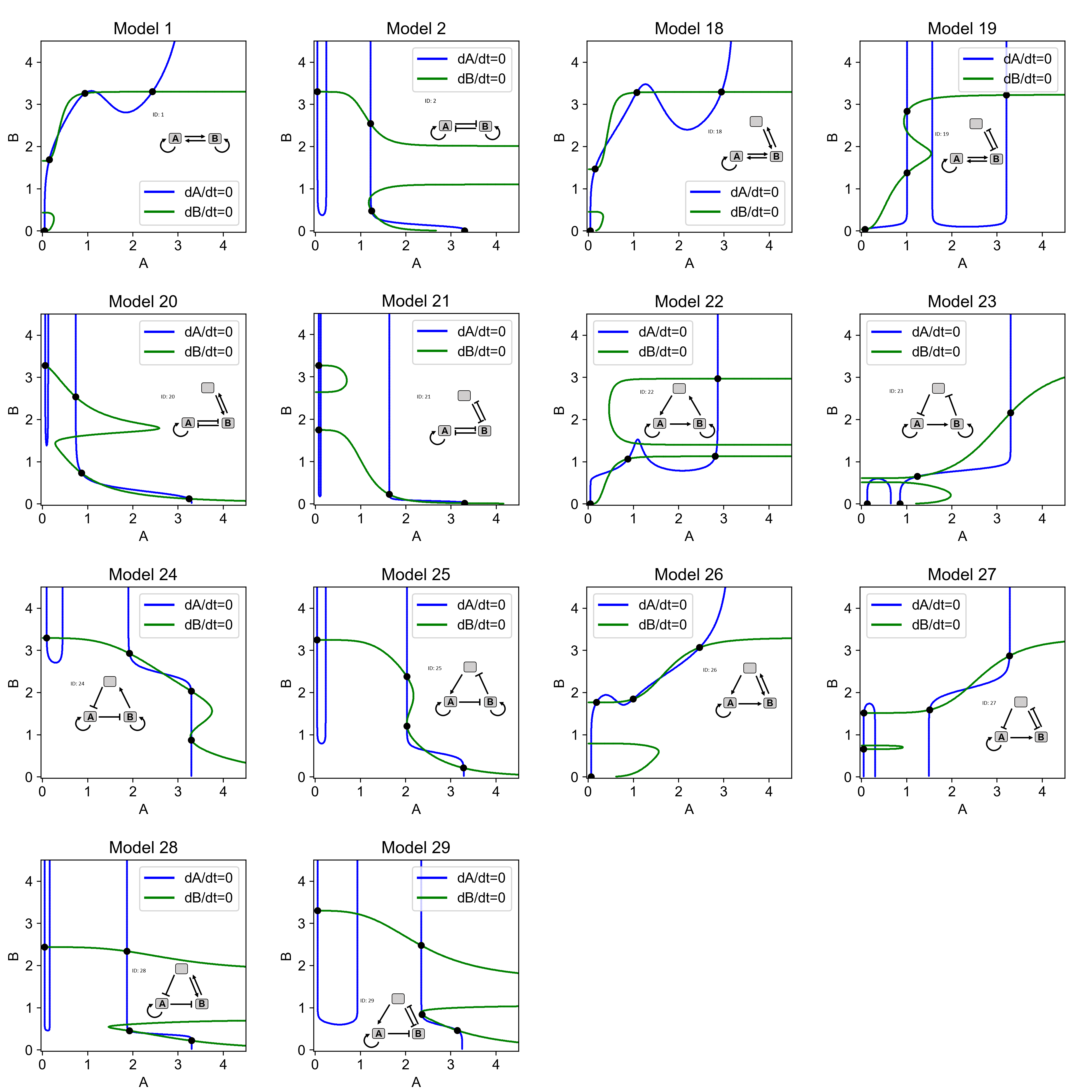

Supplement: S2 Fig — Nullclines for TF A (the node on the left of the network diagram) and TF B (the node on the right of the network diagram) are shown. Stable steady states are shown as black dots. Random parameter sampling was used to obtain the parameter sets that allows the 4-attractor systems. (TIF) [file pcbi.1006855.s008.tif]

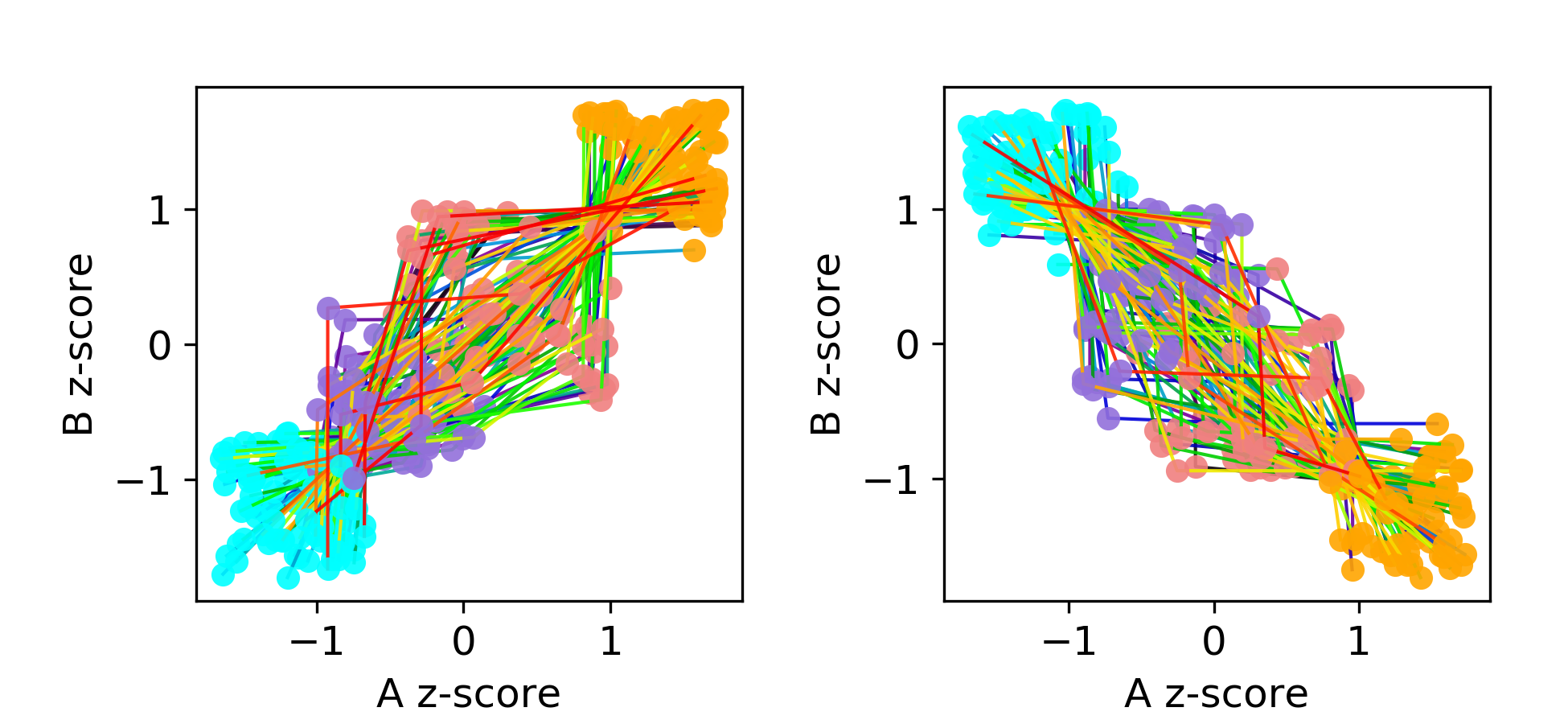

Supplement: S3 Fig — Factor A denotes the TF on the left of the network diagram. Factor B denotes the TF on the right of the network diagram. In some topologies A and B are positively correlated (left panel), whereas they are negatively correlated in other topologies (right panel). Colored dots denote the stable steady states. Colored lines connect states of their corresponding topologies. The colors of the cell states match the illustration in Fig 1. The colors of the lines denote different representative models. z-score is calculated by shifting the mean of each four attractors to 0 and then normalizing the four data points to unit variance data. All models shown in this figure are built with additive form of Hill functions. (TIF) [file pcbi.1006855.s009.tif]

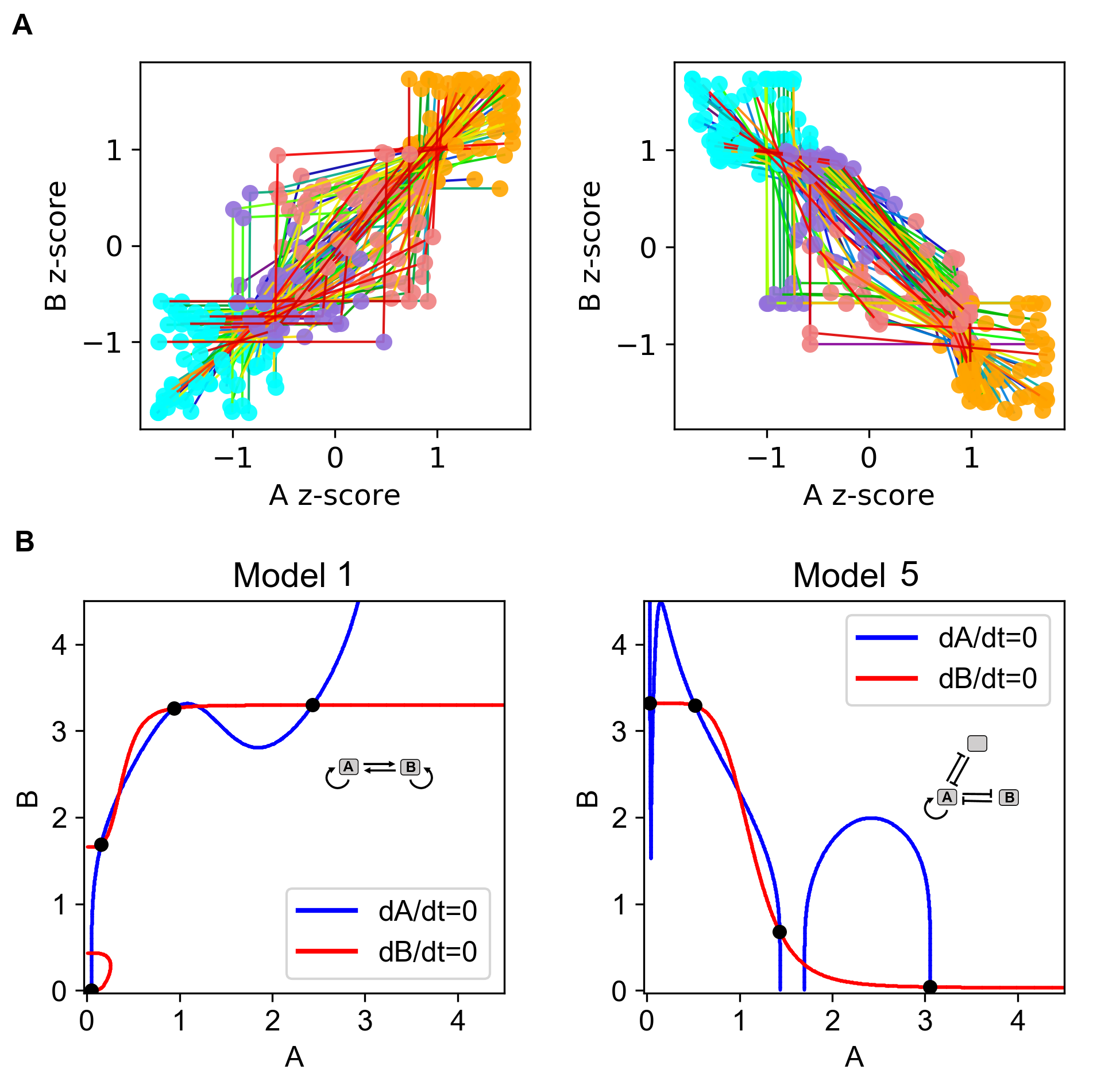

Supplement: S4 Fig — A. Overlaid four attractors for each of the 216 topologies from the 3-node network that produce 4-attractor systems. Factor A is the TF on the left of the network diagram. Factor B is the TF on the right of the network diagram. In some topologies A and B and positively correlated (left panel), whereas they are negatively correlated in other topologies (right panel). Colored dots denote the stable steady states. Colored lines connect states of their corresponding topologies. The colors of the cell states match the illustration in Fig 1. The colors of the lines denote different representative models. z-score is calculated by shifting the mean of each four attractors to 0 and then normalizing the four data points to unit variance data. B. Example phase planes for two minimum topologies (Type I and Type II respectively). In each case, four out of the seven steady states (intersections denoted by solid dots) are stable. All models shown in this figure are built with multiplicative form of Hill functions. (TIF) [file pcbi.1006855.s010.tif]

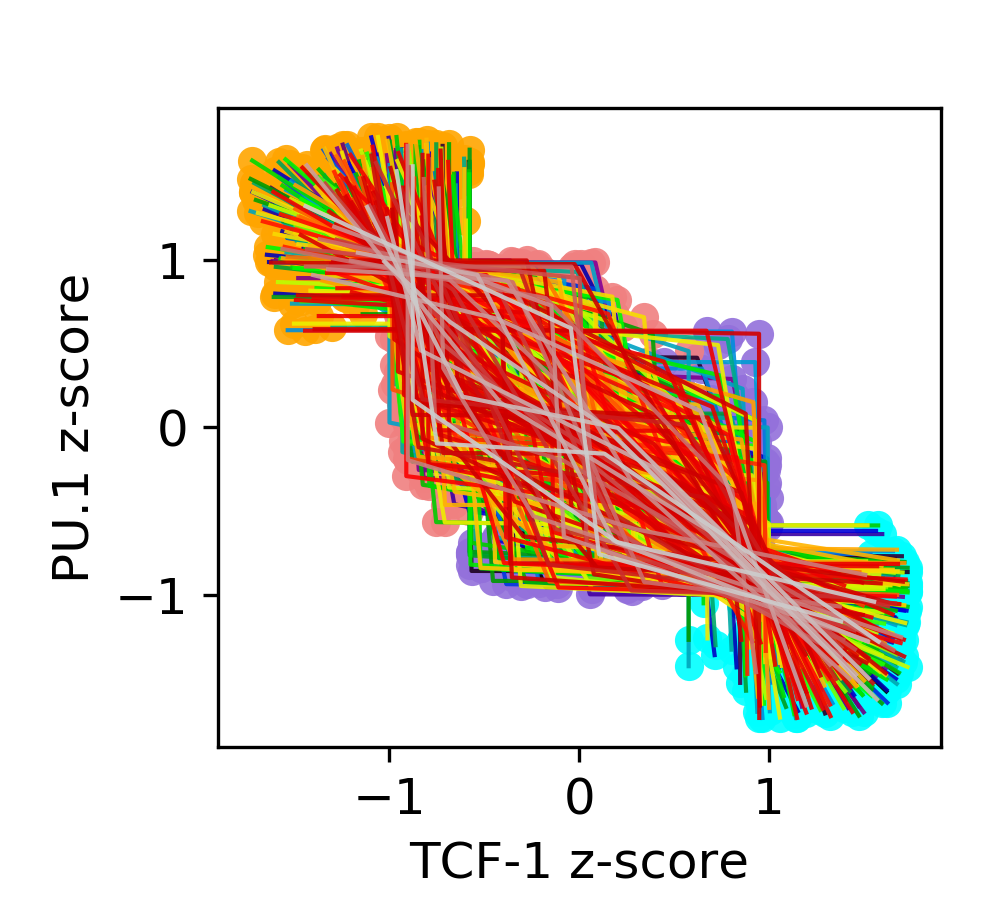

Supplement: S5 Fig — Colored dots denote the stable steady states. Colored lines connect states of their corresponding topologies. The colors of the cell states match the illustration in Fig 1. The colors of the lines denote different representative models. z-score is calculated by shifting the mean of each four attractors to 0 and then normalizing the four data points to unit variance data. All models shown in this figure are built with multiplicative form of Hill functions. (TIF) [file pcbi.1006855.s011.tif]

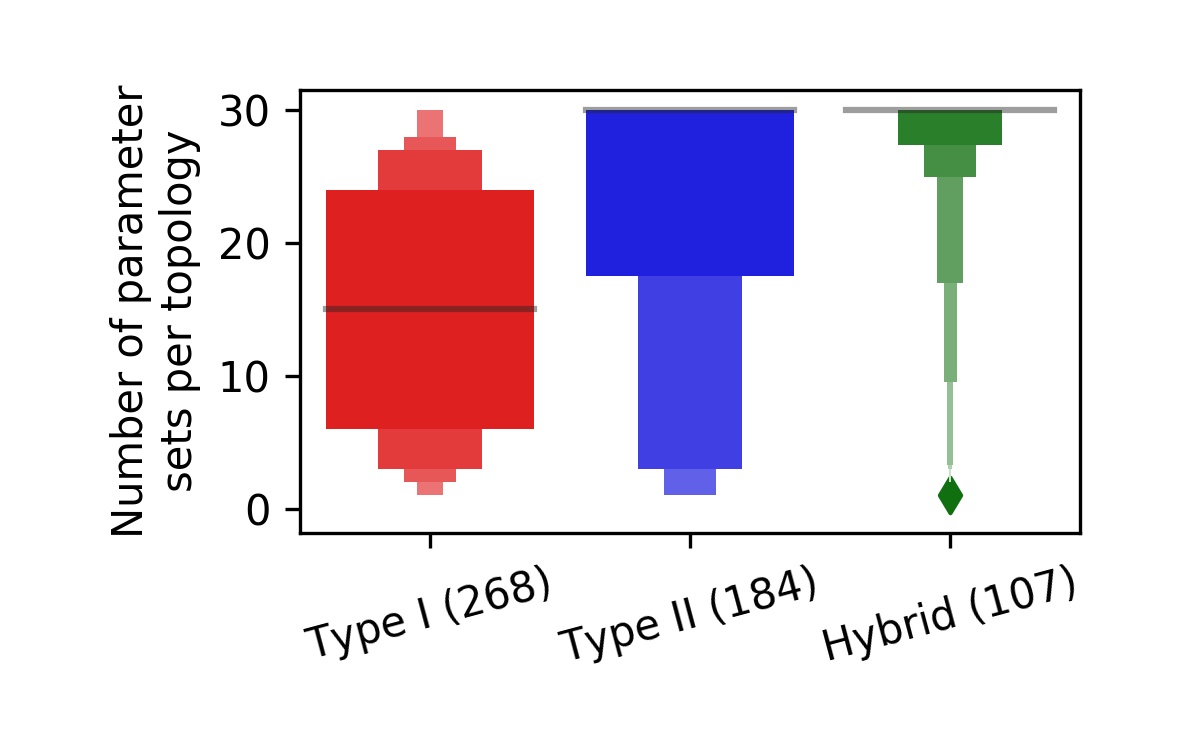

Supplement: S6 Fig — Letter-value plot shows distributions of the numbers of topologies from the entire complexity atlas (Fig 4C) over the space of parameter sets that generate the four-attractor systems per 106 random parameter sets. Distributions are separately shown for three types of motifs. Red: Type I motif. Blue: Type II motif. Green: Hybrid motif. (TIF) [file pcbi.1006855.s012.tif]

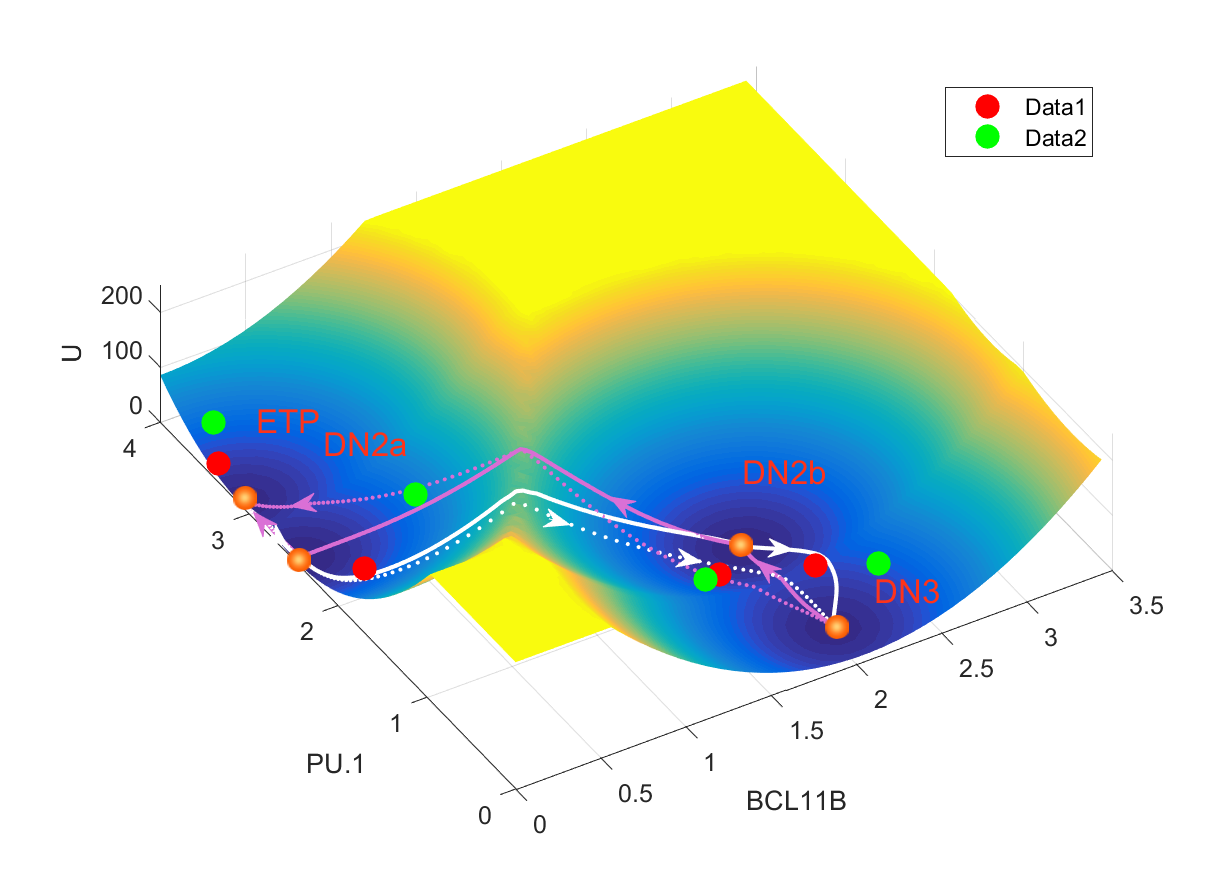

Supplement: S7 Fig — The blue regions represent higher probability or lower potential and the yellow regions represent lower probability or higher potential. Four basins of attractions characterize four different cell states (ETP, DN2a, DN2b, and DN3). White solid lines represent the MAP from ETP state to DN2a, DN2b, and DN3 states. Magenta solid lines represent the MAP from DN3 to DN2b, DN2a, and to ETP state. Dashed lines represent the direct MAP from ETP to DN3 and the direct MAP from DN3 to ETP states, respectively. The normalized gene express data (BCL11B and PU. 1) of four stages for T cell development are mapped on the landscape, where the golden balls represent the four steady states (four stages of T cell development) from the models, the red balls (Data1) represent the data from Zhang et al. [47], and the green balls represent the data from Mingueneau et al. [17]. (TIF) [file pcbi.1006855.s013.tif]

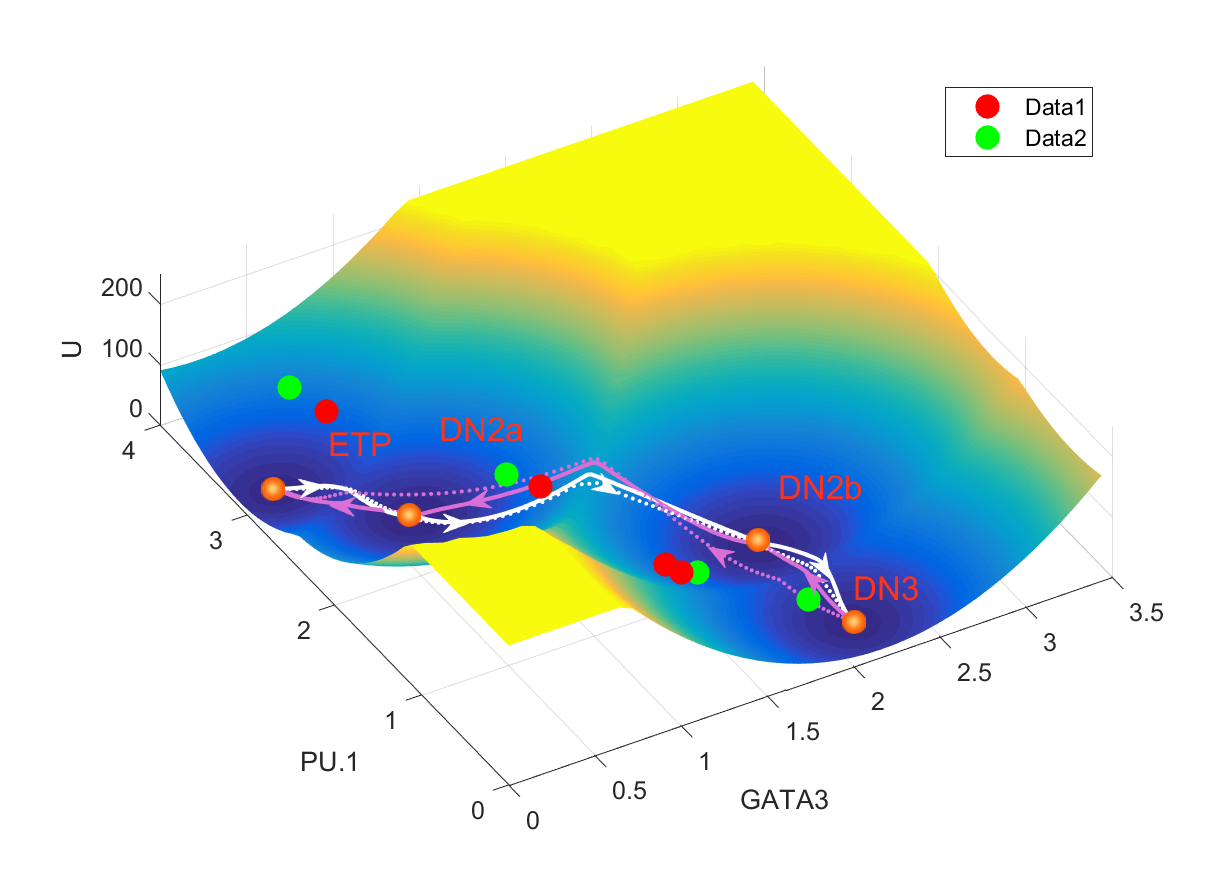

Supplement: S8 Fig — The blue regions represent higher probability or lower potential and the yellow regions represent lower probability or higher potential. Four basins of attractions characterize four different cell states (ETP, DN2a, DN2b, and DN3). White solid lines represent the MAP from ETP state to DN2a, DN2b, and DN3 states. Magenta solid lines represent the MAP from DN3 to DN2b, DN2a, and to ETP state. Dashed lines represent the direct MAP from ETP to DN3 and the direct MAP from DN3 to ETP states, respectively. The normalized gene express data (GATA3 and PU. 1) of four stages for T cell development are mapped on the landscape, where the golden balls represent the four steady states (four stages of T cell development) from the models, the red balls (Data1) represent the data from Zhang et al. [47], and the green balls represent the data from Mingueneau et al. [17]. (TIF) [file pcbi.1006855.s014.tif]

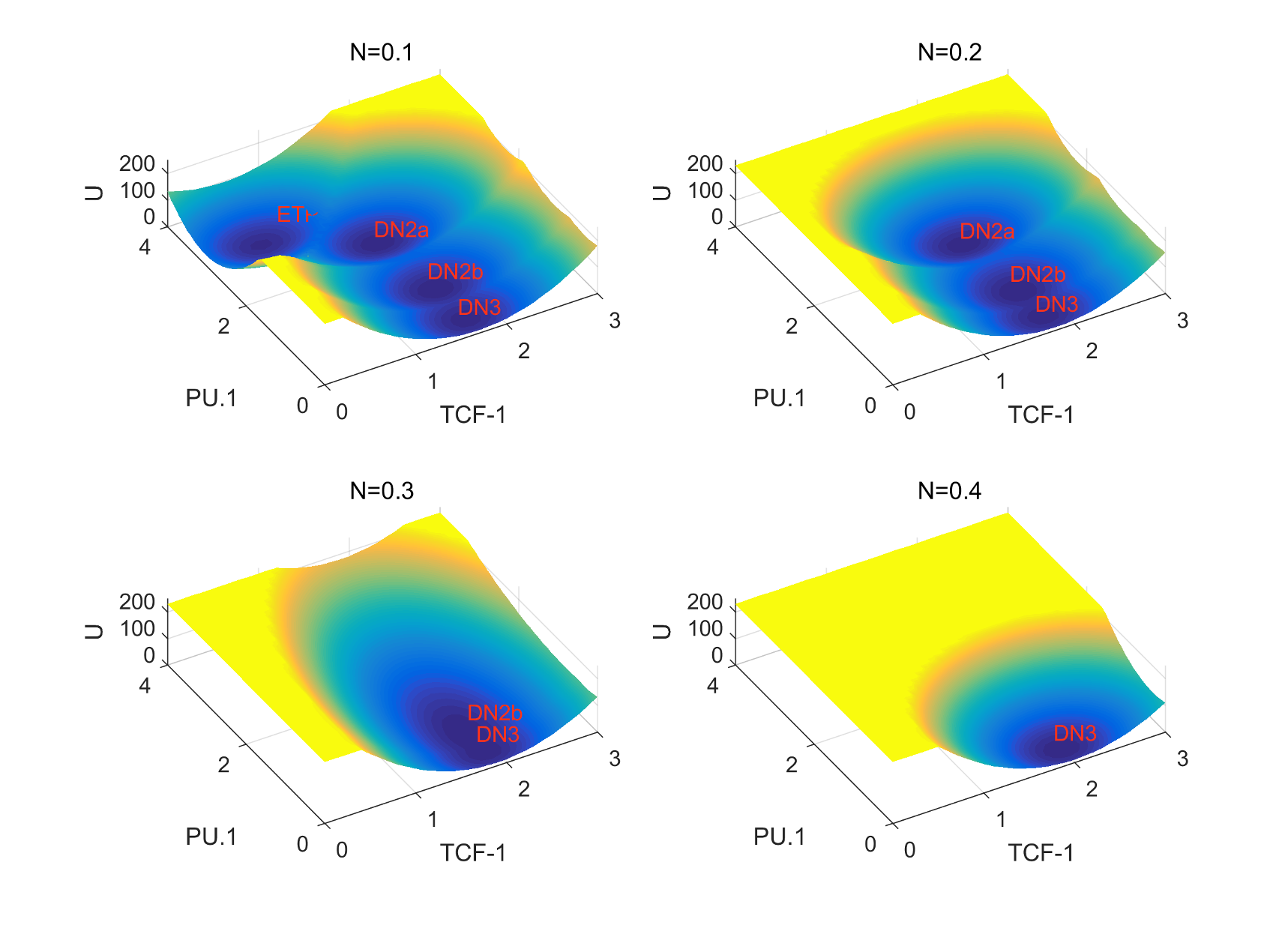

Supplement: S9 Fig — On the landscape, the blue regions represent higher probability or lower potential and the yellow regions represent lower probability or higher potential. As the Notch signal (N) increases (from 0.1 to 0.2, 0.3, and 0.4), the landscape changes from a quadristable (four stable states coexist), to tristable (DN2a, DN2b and DN3 coexist), to bistable (DN2b and DN3 coexist) and finally to a monostable DN3 state. The landscape change shows how the Notch signal promotes the transition from ETP to DN3, quantitatively. (TIF) [file pcbi.1006855.s015.tif]

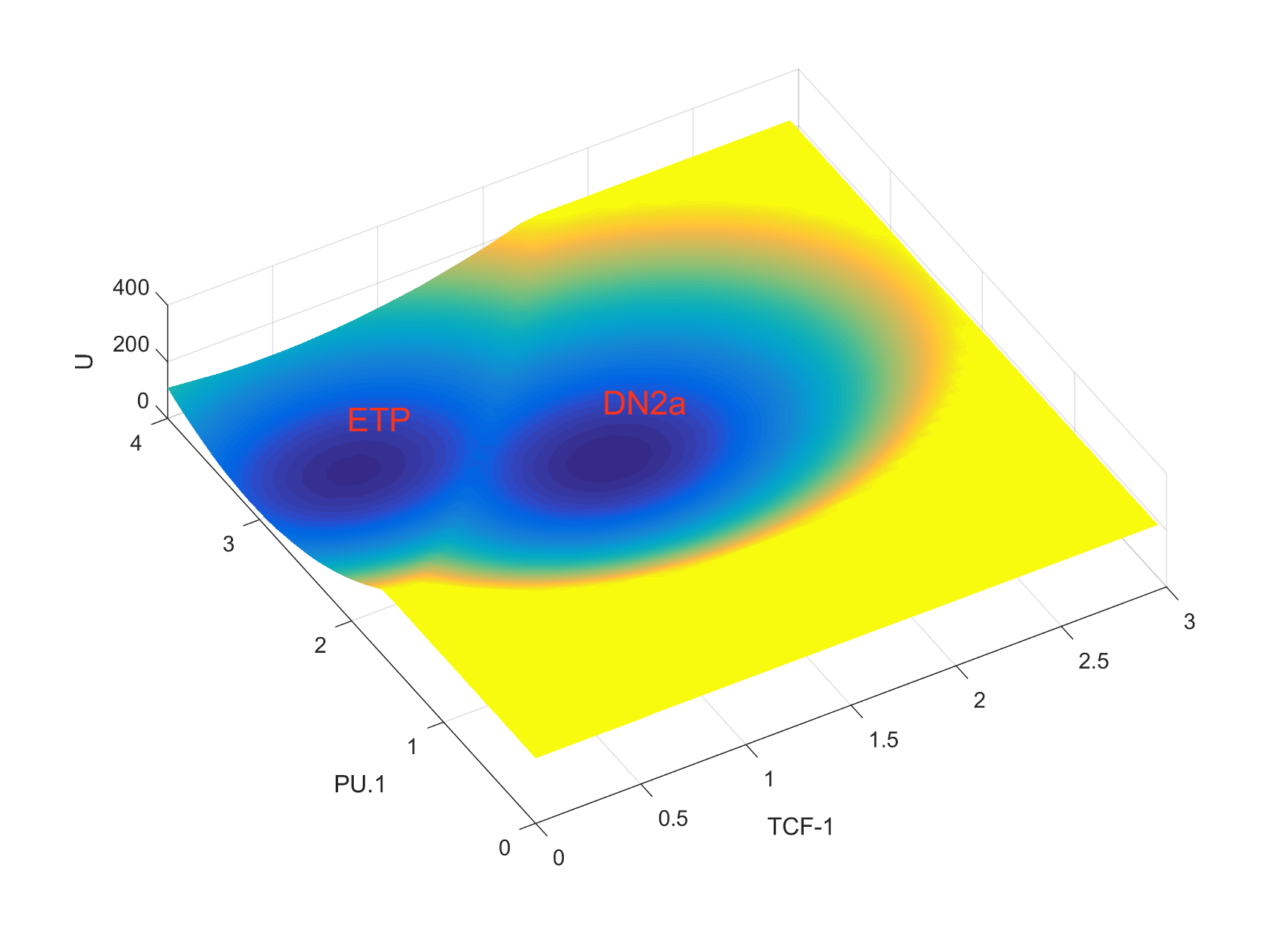

Supplement: S10 Fig — With the Bcl11b knockout (kB = 0), the landscape changes from a quadristable (four stable states coexist), to a bistable (ETP and DN2a) state. On the landscape, the blue regions represent higher probability or lower potential and the yellow regions represent lower probability or higher potential. Two basins of attractions characterize two different cell states (ETP and DN2a). The landscape change shows that the Bcl11b knockout will make ETP/DN2a states more stable and DN2b/DN3 less stable. (TIF) [file pcbi.1006855.s016.tif]

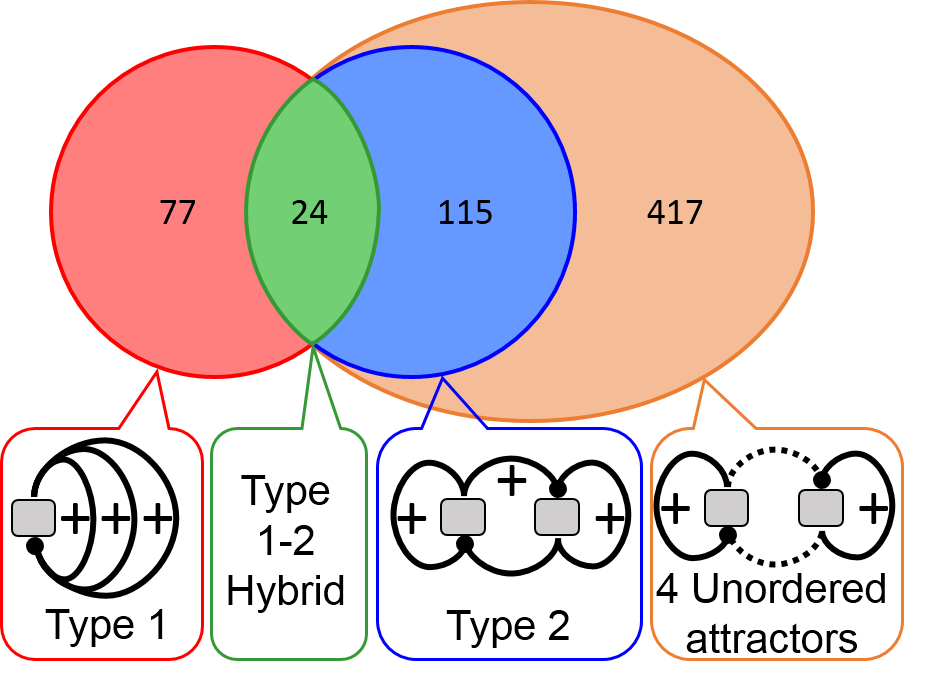

Supplement: S11 Fig — Red and blue areas correspond to Type I and Type II motifs shown in Fig 2B. Green area corresponds to motifs that contain both Type I and Type II networks. Orange area corresponds to motifs that can only produce four unordered attractors, in which the concentrations of the TFs are non-monotonically correlated. Numbers in the diagrams denote the total numbers of non-redundant topologies for each type. The Type II (blue) and Hybrid (green) motifs can produce both ordered and unordered 4-attractor systems, depending on the choice of parameters. (TIF) [file pcbi.1006855.s017.tif]

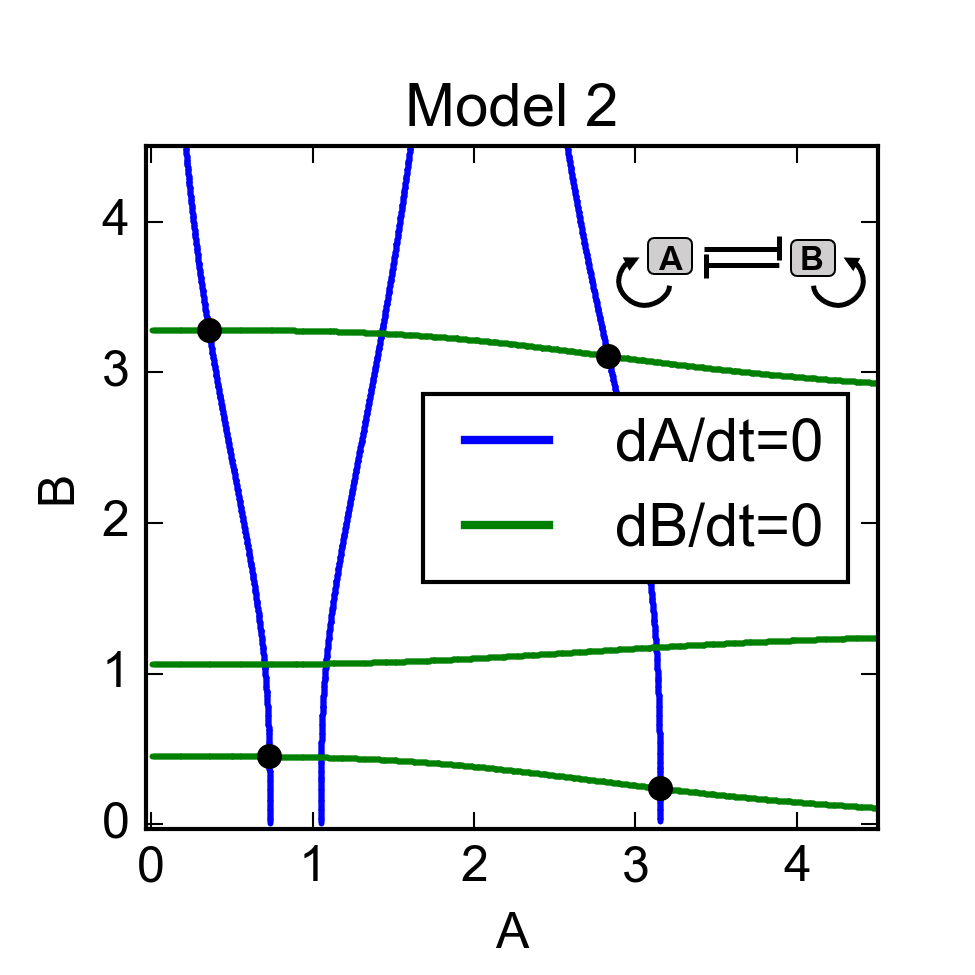

Supplement: S12 Fig — Model 2 (Type II) is chosen as an example. See S2 Fig for the scenario with ordered attractors. Nullclines for TF A (the node on the left of the network diagram) and TF B (the node on the right of the network diagram) are shown. Stable steady states are shown as black dots. Random parameter sampling was used to obtain the parameter set. (TIF) [file pcbi.1006855.s018.tif]

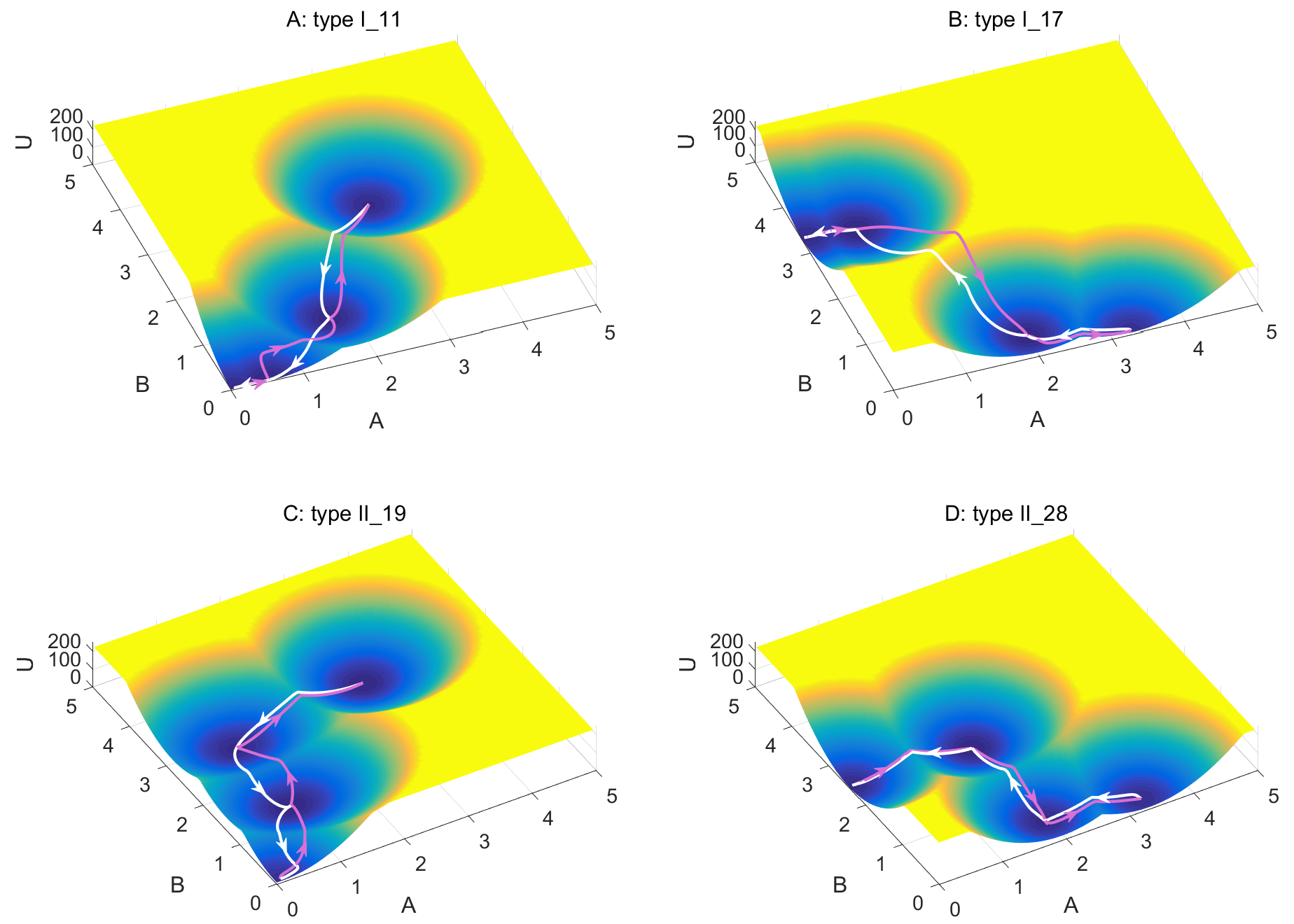

Supplement: S13 Fig — To make comparisons, two models (A and B) are picked from Type I motifs (Model 11 and Model 17 in S1 Fig) and two models (C and D) are picked from Type II motifs (Model 19 and Model 28 in S2 Fig). On the landscape, the blue regions represent higher probability or lower potential and the yellow regions represent lower probability or higher potential. For each subfigure, four basins of attractions characterize four different cell states (ETP, DN2a, DN2b, and DN3). Both Type I motif (A and B) and Type II motif (C and D) can generate stable four-attractor system. (TIF) [file pcbi.1006855.s019.tif]

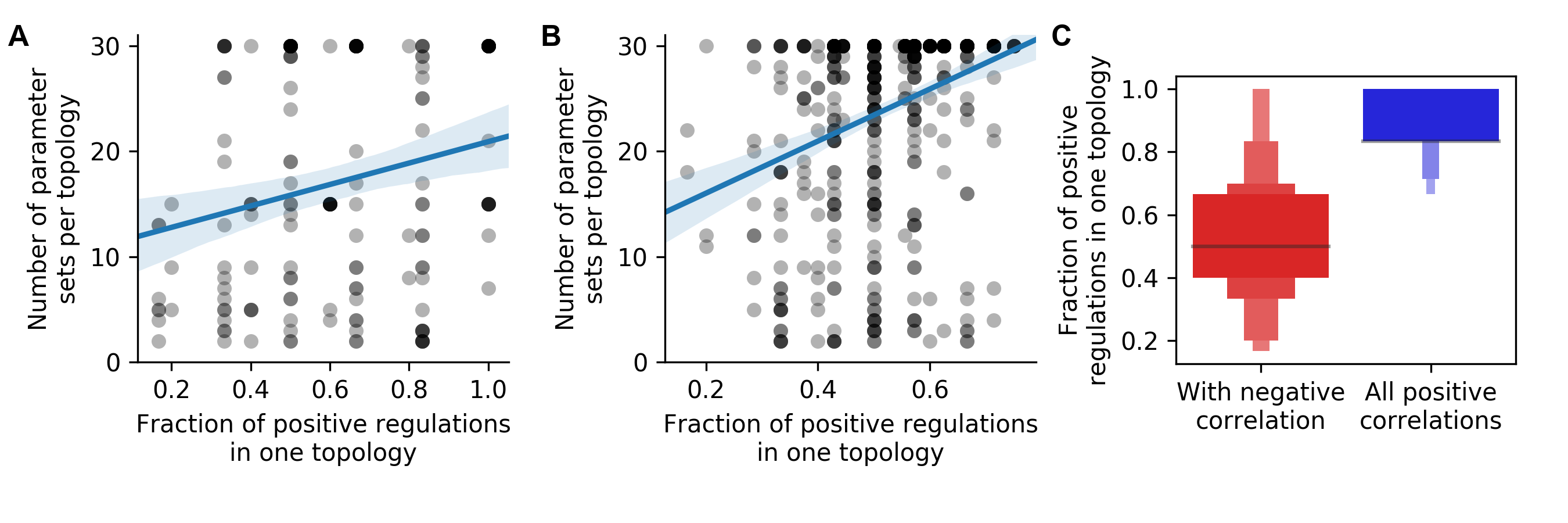

Supplement: S14 Fig — Scatter plots and regression lines show correlations of the numbers of successful parameter sets per topology and the fractions of positive regulations in corresponding topologies. A. Sampled 3-node generic topologies. Spearman correlation coefficient: 0.21, p-value = 0.008. B. Subnetworks of the T cell development model. Spearman correlation coefficient: 0.31, p-value < 0.0001. C. Comparison of networks generating attractors with all positively correlated TFs and those with at least one pair of inversely correlated TFs. Three-node generic topologies were analyzed. (TIF) [file pcbi.1006855.s020.tif]

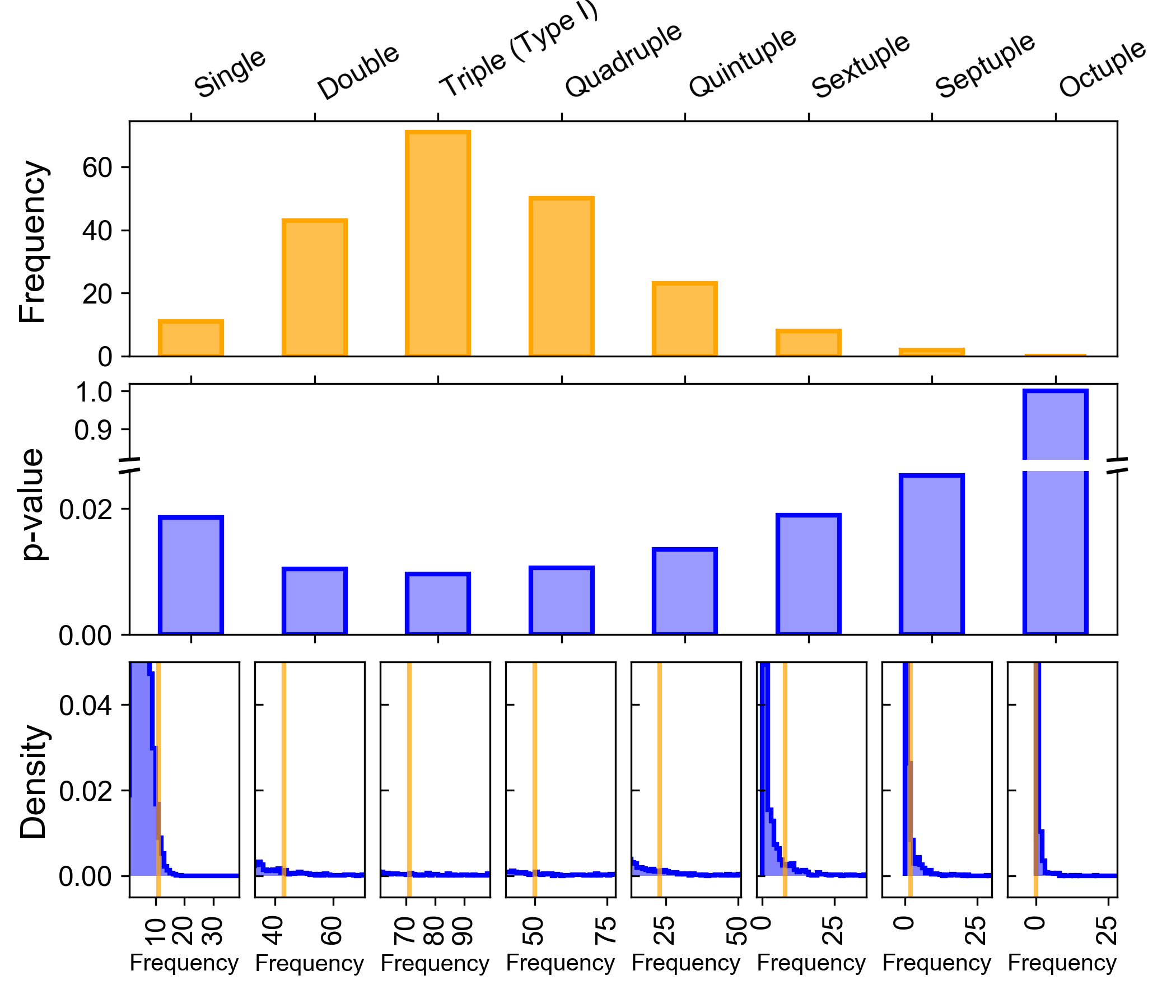

Supplement: S15 Fig — Top panel: total occurrences of various types of motifs in the T cell network. Middle panel: empirical p-values (middle panel) for these motifs with a background network population. Bottom panel: an illustration of the p-values with the distributions of the background population. Each motif has n (0<n<9) positive feedback loops, all of which share at least one TFs in the motif. Type I motif is a special case of such motifs with n = 3. Random networks were obtained by assigning random regulations (positive, negative or none) between each pair of TFs. 105 random networks were generated. Empirical p-values were obtained by counting the number of the random networks with the motifs not less than those in the T cell network. See Methods for details of the p-value definition. Distributions of motif frequencies obtained from the random networks are shown in the bottom panel. The yellow vertical bars represent the number of occurrences in the T cell network. The right-tail areas defined by the vertical bars correspond to the p-values shown in the middle panel (blue bars). (TIF) [file pcbi.1006855.s021.tif]

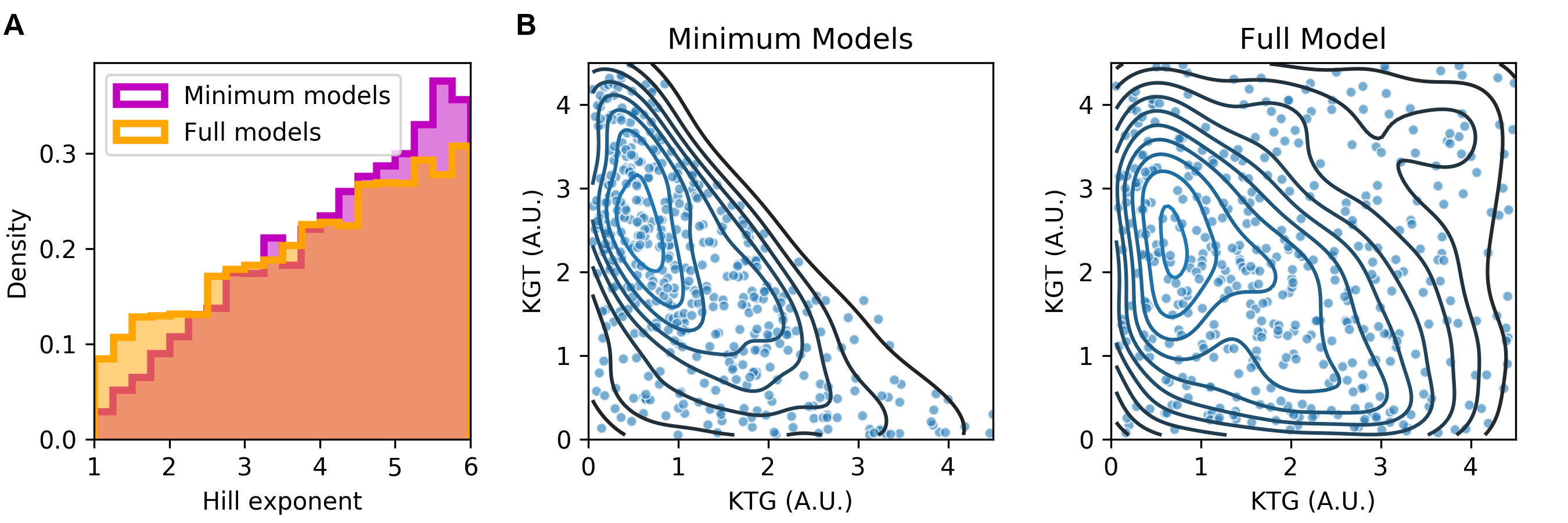

Supplement: S16 Fig — A. Distributions of Hill exponents (steepness of regulations) of 900 parameter sets from each category. The parameter sets from 66 minimum motifs are combined. B. Distributions of the activation threshold parameters of the regulation of GATA3 by TCF-1 and the regulation of TCF-1 by GATA3. 440 parameter sets (maximum number of parameter sets from minimum motifs that contain these two parameters) from each category are plotted. (TIF) [file pcbi.1006855.s022.tif]
